# Oxidative 3,3,3-trifluoropropylation of arylaldehydes

**DOI:** 10.3762/bjoc.9.279

**Published:** 2013-11-11

**Authors:** Akari Ikeda, Masaaki Omote, Shiho Nomura, Miyuu Tanaka, Atsushi Tarui, Kazuyuki Sato, Akira Ando

**Affiliations:** 1 Faculty of Pharmaceutical Sciences, Setsunan University, 45–1, Nagaotoge-cho, Hirakata, Osaka 573–0101, Japan

**Keywords:** cesium fluoride, organo-fluorine, 1,3-proton shift, trifluoromethyl, 3,3,3-trifluoropropenyl

## Abstract

A reaction between (*E*)-trimethyl(3,3,3-trifluoroprop-1-en-1-yl)silane (**1**) and arylaldehydes **2** was triggered by fluoride anions to afford aryl 3,3,3-trifluoropropyl ketones **3** in moderate to good yield. A mechanistic study of this reaction indicated that it occurred via an allyl alkoxide (**4**). A subsequent 1,3-proton shift of the benzylic proton of **4** forms **3**. This reaction involves oxidative 3,3,3-trifluoropropylation of an arylaldehyde to afford 4,4,4-trifluoro-1-arylbutan-1-one.

## Introduction

Trifluoromethyl groups are an essential motif in pharmaceuticals, agricultural chemicals, and functional materials because trifluoromethylation of such chemicals often significantly improves their performance [1–6]. To date, the trifluoromethylation of carbonyl compounds [7–10] and aryl halides [11–13] has been extensively explored. On the other hand, the 2,2,2-trifluoroethylation and the 3,3,3-trifluoropropylation, which elongate the product by two and three carbon atoms, respectively, have not been explored yet. 2-Bromo-3,3,3-trifluoropropene [14–26] and 1,1,1,3,3-pentafluoropropane [27–28] have been used in place of (3,3,3-trifluoropropynyl)lithium, which can add to carbonyl compounds and couple with aryl halides through a zinc intermediate. For the 3,3,3-trifluoropropenyl synthon Yamazaki et al. reported the use of 2-(trifluoromethyl)-1-(phenylsulfenyl)vinyltrimethylsilane for the addition to aldehydes in the presence of fluoride anion [29]. Recently, Prakash et al. [30] reported the synthesis of β-trifluoromethylstyrenes through a Heck coupling reaction of aryl iodides with 1-iodo-3,3,3-trifluoropropane, allowing a direct introduction of 3,3,3-trifluoropropenyl groups to aromatic rings. Approximately at the same time, our research group demonstrated that (*E*)-trimethyl(3,3,3-trifluoroprop-1-en-1-yl)silane (**1**) effectively participated in a Hiyama cross-coupling reaction with aryl iodide to construct β-trifluoromethylstyrenes in good to excellent yield [31–32]. In the course of this study, we found that the reaction of **1** with benzaldehyde in the presence of fluoride anions afforded an allyl alcohol, which spontaneously isomerized to phenyl 3,3,3-trifluoropropyl ketone (**3a**). This reaction involves a one-pot synthesis of aryl 3,3,3-trifluoropropyl ketones from the corresponding arylaldehyde, representing the oxidative 3,3,3-propenylation of the arylaldehyde. Here, we report our study of this unusual reaction.

## Results and Discussion

At the outset of our study, we attempted selective C–Si bond dissociation of **1** with several fluoride anion sources. The subsequently generated carbanion was trapped with benzaldehyde to generate 4,4,4-trifluoromethylallyl alcohol **4** (Table 1). We found that this reaction did not take place when either TiF_4_ in THF or CuF_2_/dppp in DMF was used (Table 1, entries 1 and 2). However, the reaction proceeded when two equivalents of CsF to arylaldehyde **2** were used in DMA at a temperature of 55 °C, affording **3a** and **4a** in 15% and 16% yield, respectively (Table 1, entry 3). In contrast, the reaction did not proceed in THF (Table 1, entry 4). The yield increased as the temperature was raised to 80 °C, but a further increase to 100 °C did not improve the yield of the reaction (Table 1, entries 5 and 6). Changing the solvent to DMF considerably shortened the reaction time. The added amount of CsF also influenced this reaction. The reaction conducted with four equivalents of CsF afforded **3a** and **4a** in 52% and 27% yield, respectively (Table 1, entry 8). However, a further increase of the amount of CsF did not improve the reaction yield (Table 1, entry 9). We thus determined that the conditions used in entry 8 of Table 1 were optimal. We explored the scope and limitation of this reaction by using these optimal conditions.

**Table 1:** Reaction of **1** with benzaldehyde in the presence of fluoride anions.^a^

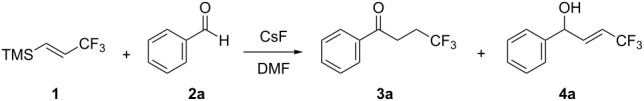							

entry	F anion (equiv)^b^	**1** (equiv)^b^	solvent	temp (°C)	time (h)	yield (%)^c,d^	
						**3a**	**4a**

1	TiF_4_ (2)	2	THF	60	24	–	–
2	CuF_2_ (1)/dppp (2)	2	DMF	80	26	–	–
3	CsF (2)	1.5	DMA	55	24	15	16
4	CsF (2)	1.5	THF	55	24	–	–
5	CsF (2)	2	DMA	80	4	53	–
6	CsF (2)	2	DMA	100	4	47	–
7	CsF (2)	2	DMF	80	1	49	10
8	CsF (4)	2	DMF	80	1	52 (43)	27
9	CsF (6)	2	DMF	80	1	38	5

^a^The reaction was carried out with **2a** (0.2 mmol) and a solvent (2.0 mL). ^b^The value in parentheses indicates equiv with respect to benzaldehyde (0.2 mmol). ^c^NMR yields, which were calculated by integration of the ^19^F NMR signals of **3a** and **4a** relative to that of the internal standard of 1,4-bis(trifluoromethyl)benzene. ^d^The value in parentheses indicates isolated yield (%).

A variety of arylaldehydes participated in the reaction to give products in moderate to good yields (Table 2). The reactions with benzaldehyde derivatives with an electron-withdrawing group, such as chloro-, bromo-, fluoro- or trifluoromethyl, at the *para*-position proceeded to give **3b**–**e** in moderate to good yields (Table 2, entries 1 to 4). The substitution with other electron-withdrawing groups such as methoxycarbonyl and cyano decreased the yield of the product, **3f** and **3g** were obtained in 38% and 46% yield, respectively (Table 2, entries 5 and 6). In contrast, the substitution of benzaldehyde with an electron-donating group slowed down the reaction substantially. *p*-Methyl and *p*-methoxybenzaldehydes gave **3h** and **3i** in 26% and 17% yield, respectively (Table 2, entries 7 and 8). *meta*-Substitution of benzaldehyde with a methoxy group gave **3j** in 58% yield (Table 2, entry 9). These results are in accordance with the Hammett equation. *ortho*-Substitution of substrates decreased the yield of the products substantially, probably because of the steric hindrance introduced by the substituent (Table 2, entries 10 and 11). While the reaction could be applied to polycyclic and heterocyclic compounds, **3m** and **3n** were only obtained in low yield (Table 2, entries 12 and 13). An aliphatic aldehyde did not participate in the reaction (Table 2, entry 14).

**Table 2:** Investigation of reaction scope with optimal conditions.^a^

entry	Ar–CHO	**3**	yield (%)^b^	**4**	yield (%)^b^

1	*p*-Cl-C_6_H_4_	**3b**	72	**4b**	8
2	*p*-Br-C_6_H_4_	**3c**	79	**4c**	6
3	*p*-F-C_6_H_4_	**3d**	55	**4d**	4
4	*p*-CF_3_-C_6_H_4_	**3e**	59	**4e**	6
5	*p*-CH_3_OC(O)-C_6_H_4_	**3f**	38	**4f**	6
6	*p*-CN-C_6_H_4_	**3g**	46	**4g**	10
7	*p*-CH_3_-C_6_H_4_	**3h**	26	**4h**	8
8	*p*-CH_3_O-C_6_H_4_	**3i**	17	**4i**	16
9	*m*-CH_3_O-C_6_H_4_	**3j**	58	**4j**	10
10	*o*-CH_3_O-C_6_H_4_	**3k**	12	**4k**	35
11	*o*-Cl-C_6_H_4_	**3l**	16	**4l**	16
12	2-naphthyl	**3m**	41	**4m**	5
13	3-pyridyl	**3n**	31	**4n**	12
14	C_6_H_5_(CH_2_)_2_	**3o**	–	**3o**	–

^a^Reactions were carried out with **1** (0.4 mmol), **2** (0.2 mmol) and CsF (0.8 mmol) in DMF (2.0 mL) at 80 °C. ^b^NMR yields, which were calculated by integration of the ^19^F NMR signals **3** and **4** relative to that of the internal standard of 1,4-bis(trifluoromethyl)benzene.

**Scheme 1:**
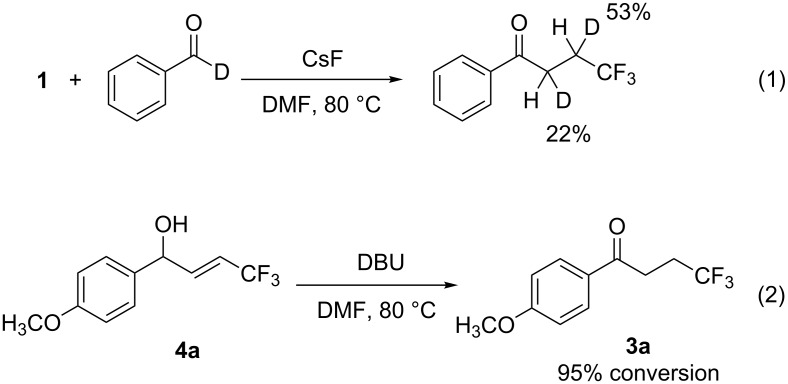
Experiments to elucidate the reaction mechanism.

**Scheme 2:**
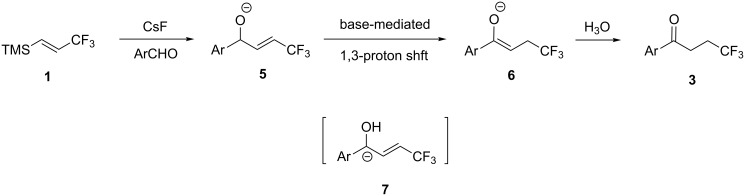
The proposed reaction mechanism.

During the course of this study, it became obvious that the reaction gave allyl alcohol **4** first, and then subsequent transformation of **4** into **3** occurred during the reaction process. Recently, both Cahard et al. [33–34] and Qing et al. [35] independently reported that the isomerization of a 4,4,4-trifluoromethylallyl alcohol substructure was promoted by a ruthenium catalyst to form a 3,3,3-trifluoropropylcarbonyl unit. We hypothesized that the formation of **3** would be promoted by inter- or intramolecular migration of the benzylic proton of **4** in the basic medium. To confirm the hypothesis, we conducted the reaction by using benzaldehyde-*d* to clarify whether or not proton migration was involved in the reaction sequence. When benzaldehyde-*d* was treated with **1** and CsF in DMF at 80 °C, the reaction gave a product in which deuterium was inserted on the α- and β-carbons of the carbonyl group with rates of 22% and 53%, respectively (Scheme 1, (1)). This result suggests that deuterium on the β-carbon should be incorporated by a 1,3-proton shift. In contrast, deuterium on the α-carbon should be inserted when the enolate intermediate, which is generated during the reaction, intermolecularly extracted the benzylic deuterium of **4**. To further investigate the reaction mechanism, **4a** was exposed to basic conditions to observe whether or not the isomerization of **4a** to **3a** would occur. Indeed, the treatment of **4a** with a stoichiometric amount of DBU resulted in an almost quantitative conversion to **3a** (Scheme 1, (2)).

This suggests that the reaction provides **4** first. **4** subsequently isomerizes to form **3**, which is an oxidative 3,3,3-trifluoropropylation of the arylaldehyde. These results allowed us to propose the reaction mechanism shown in Scheme 2, which includes the isomerization of **5** to **6** in the formation of **3**. To support our proposed mechanism, a computational calculation was performed with Gaussian 03W at the B3LYP/6-31+G* level of theory to confirm that key intermediate **7** was involved in the isomerization process. The calculation indicated that intermediate **5** generated in the first step of the reaction was slightly more stable (0.417 kcal/mol) than **7**. The slight energy difference between **5** and **7** indicates the viability of **7** in the reaction medium. The generation of **7** would facilitate the isomerization of **5** to **6**.

## Conclusion

We reacted **1** with benzaldehyde in the presence of CsF to provide **3**. We demonstrated that **4** was generated in the first step of the reaction, and then subsequently isomerized to **3** under basic conditions. This sequential reaction involves an oxidative 3,3,3-trifluoropropylation of the arylaldehyde to form a 3,3,3-trifluoropropylcarbonyl unit. A further refinement of the reaction conditions to enhance the compatibility of the reaction with substrates such as aliphatic aldehydes is in progress in our group.

## Supporting Information

File 1 Experimental details and characterization data for all new compounds.
