# Supplementary material for: Oxidative 3,3,3-trifluoropropylation of arylaldehydes
Source: Beilstein J Org Chem. 2013 Nov 11;9:2417–21. doi: 10.3762/bjoc.9.279 (PMC3869248; doi:10.3762/bjoc.9.279)
Supplement: File 1 — Experimental details and characterization data for all new compounds. [file Beilstein_J_Org_Chem-09-2417-s001.pdf]

Supporting Information

for

Oxidative 3,3,3-trifluoropropylation of arylaldehydes

Akari Ikeda, Masaaki Omote, Shiho Nomura, Miyuu Tanaka, Atsushi Tarui, Kazuyuki Sato and Akira Ando*

Address: Faculty of Pharmaceutical Sciences, Setsunan University, 45–1, Nagaotoge-cho, Hirakata, Osaka 573–0101, Japan

Email: Akira Ando - aando@pharm.setsunan.ac.jp

*Corresponding author

Experimental details and characterization data for all new compounds

General information …… S2
Experimental section …… S2
Typical procedure for the reaction of **1** with aryl aldehyde ……S2
Isomerization reaction of **4**…… S2
Spectroscopic data …… S3–S6
Structures of **5** and **7** obtained from computational calculation……S7

General information:

General information. All experiments were carried out under an argon atmosphere in flame-dried glassware using standard inert techniques for introducing reagents and solvents, unless otherwise noted. *N*,*N*-Dimethylformamide (DMF) and *N*,*N*-dimethylacetamide (DMA) was distilled over calcium hydride and stored in a bottle with activated molecular sieves (4 Å). Tetrahydrofuran (THF) was distilled over benzophenone ketyl sodium just before use. All commercially available materials were used as received without further purification. ^{1}H and ^{13}C NMR spectra were recorded at room temperature on a commercial measurement device at 400 and 600 MHz. A ^{19}F NMR spectrum was recorded at room temperature on a commercial measurement device at 90 and 600 MHz. Chemical shifts of ^{1}H NMR and ^{13}C NMR are reported in parts per million from tetramethylsilane (TMS), used as an internal standard. Chemical shifts of ^{19}F NMR are reported in parts per million from $CFCl_3$, used as an internal standard. All data are reported as follows: chemical shifts, relative integration value, multiplicity (s = singlet, d = doublet, t = triplet, q = quartet, sep = septet, br = broad, brd = broad-doublet, m = multiplet), and coupling constants *J* (Hz). High-resolution mass spectrometry (HRMS) experiments were performed with a double-focusing mass spectrometer with EI ionization. Infrared (IR) spectra were recorded in KBr disks or thin films on KBr plates. Melting points are not corrected.

Typical procedure for the reaction of 1 with arylaldehyde

In a glove box purged with argon gas, CsF (0.4 mmol) were placed in a flask. To the flask was added anhydrous DMF (4.0 mL) and arylaldehyde **2** (0.2 mmol). The mixture was heated at 80 ºC and **1** (2 mmol) dissolved in DMF (2.0 mL) was added to the mixture over 30 minutes. The mixture was stirred at 80 ºC for one hour and then the mixture was poured into aqueous 10% HCl. The mixture was extracted with $CHCl_3$ and the $CHCl_3$ layer was dried over anhydrous $MgSO_4$. After filtration of a solid, the solvent was removed in vacuo and the residue was purified by silica gel column chromatography to give **3**.

Isomerization reaction of 4

To the flask was added **4** (0.1 mmol), DBU (0.13 mmol) and DMF (2.0 mL). The whole mixture was heated at 80 °C for two hours. The mixture was poured into aqueous 10% HCl. The mixture was extracted with $CHCl_3$ and the $CHCl_3$ layer was dried over anhydrous $MgSO_4$. After filtration of a solid, the solvent was removed in vacuo. The residue was subjected to ^{19}F NMR analysis to determine the conversion (%) of **4** to **3**, using 1,4-bis(trifluoromethyl)benzene as the internal standard, to be found that 95% of the conversion was occurred.

4,4,4-Trifluoro-1-phenylbutan-1-one (3a)

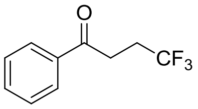

43% Yield; ^{1}H NMR ($CDCl_3$) δ 2.60 (2H, qt, J = 10.8 Hz, 7.8 Hz), 3.25-3.29 (2H, m), 7.50 (2H, t, J = 7.8 Hz), 7.61 (1H, t, J = 7.4 Hz), 7.98 (2H, d, J = 7.4 Hz); ^{13}C NMR ($CDCl_3$) δ 28.4 (q, J = 29.6 Hz), 31.2 (q, J = 2.5 Hz), 127.1 (q, J = 275.0 Hz), 128.0, 128.7, 133.6, 136.1, 196.3; ^{19}F NMR ($CDCl_3$) δ -67.25 (t, J = 11.0 Hz); MS m/z 202 (M^+); HRMS calcd for $C_{10}H_9F_3O$ 202.0605 (M^+), found 202.0600; IR (KBr) cm^{-1} 1686.44.

1-(4-Chlorophenyl)-4,4,4-trifluorobutan-1-one (3b)

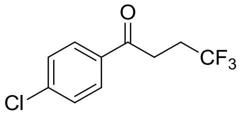

^{1}H NMR ($CDCl_3$) δ 2.59 (2H, qt, J = 10.8 Hz, 7.8 Hz), 3.21-3.25 (2H, m), 7.47 (2H, d, J = 8.8 Hz), 7.92 (2H, d, J = 8.8 Hz); ^{13}C NMR ($CDCl_3$) δ 28.4 (q, J = 29.7 Hz), 31.3 (q, J = 2.5 Hz), 127.0 (q, J = 275.2 Hz), 129.1, 129.3, 134.3, 140.1; ^{19}F NMR ($CDCl_3$) δ -67.25 (t, J = 11.0 Hz); MS m/z 236 (M^+); HRMS calcd for $C_{10}H_8ClF_3O$ 236.0216 (M^+), found 236.0208; IR (KBr) cm^{-1} 1686.44.

1-(4-Bromophenyl)-4,4,4-trifluorobutan-1-one (3c)

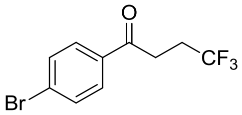

^{1}H NMR ($CDCl_3$) δ 2.59 (2H, qt, J = 10.9 Hz, 7.8 Hz), 3.21-3.25 (2H, m), 7.64 (2H, d, J = 8.3 Hz), 7.84 (2H, d, J = 8.7 Hz); ^{13}C NMR ($CDCl_3$) δ 28.4 (q, J = 29.7 Hz), 31.3 (q, J = 2.5 Hz), 127.0 (q, J = 274.9 Hz), 128.8, 129.4, 132.1, 134.7, 195.1; ^{19}F NMR ($CDCl_3$) δ -67.25 (t, J = 11.0 Hz); MS m/z 280 (M^+); HRMS calcd for $C_{10}H_8BrF_3O$ 279.9711 (M^+), found 279.9705; IR (KBr) cm^{-1} 1687.41.

4,4,4-Trifluoro-1-(4-fluorophenyl)butan-1-one (3d)

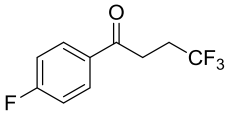

^{1}H NMR ($CDCl_3$) δ 2.61 (2H, qt, J = 10.9 Hz, 7.8 Hz), 3.23-3.27 (2H, m), 7.18 (2H, dd, J = 8.7 Hz, 8.7 Hz), 8.02 (2H, dd, J = 7.5 Hz, 5.1 Hz); ^{13}C NMR ($CDCl_3$) δ 28.5 (q, J = 29.8 Hz), 31.2 (q, J = 2.5 Hz), 115.9 (d, J = 21.7 Hz), 127.0 (q, J = 275.1 Hz), 130.6 (d, J = 8.8 Hz), 132.6 (d, J = 3.3Hz), 165.9 (d, J = 256.3 Hz), 194.5; ^{19}F NMR ($CDCl_3$) δ -67.25 (t, J = 11.0 Hz), -105.16 (m); MS m/z 220 (M^+); HRMS calcd for $C_{10}H_8F_4O$ 220.0511 (M^+), found 220.0515; IR (KBr) cm^{-1} 1685.48.

4,4,4-Trifluoro-1-(4-(trifluoromethyl)phenyl)butan-1-one (3e)

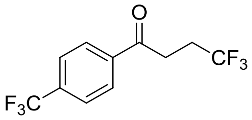

^{1}H NMR ($CDCl_3$) δ 2.62 (2H, qt, J = 10.7 Hz, 7.8 Hz), 3.27-3.31 (2H, m), 7.77 (2H, d, J = 8.3 Hz), 8.09 (2H, d, J = 7.8 Hz); ^{13}C NMR ($CDCl_3$) δ 28.3 (q, J = 29.8 Hz), 31.7 (q, J = 2.5 Hz), 123.4 (q, J = 272.9 Hz), 125.8 (q, J = 3.3 Hz), 126.9 (q, J = 275.2 Hz), 134.9 (q, J = 32.5 Hz), 138.6, 195.2; ^{19}F NMR ($CDCl_3$) δ -64.03, -67.27 (t, J = 11.0 Hz); MS m/z 270 (M^+); HRMS calcd for $C_{11}H_8F_6O$ 270.0479 (M^+), found 270.0480; IR (KBr) cm^{-1} 1698.98.

Methyl 4-(4,4,4-trifluorobutanoyl)benzoate (3f)

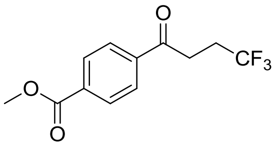

^{1}H NMR ($CDCl_3$) δ 2.61 (2H, qt, J = 10.8 Hz, 7.7 Hz), 3.27-3.31 (2H, m), 3.96 (3H, s), 8.03 (2H, d, J = 8.3 Hz), 8.16 (2H, d, J = 8.8 Hz); ^{13}C NMR ($CDCl_3$) δ 28.3 (q, J = 29.8 Hz), 31.7 (q, J = 2.5 Hz), 56.6, 126.9 (q, J = 275.1 Hz) ,127.9, 129.9, 134.3, 139.1, 165.9, 195.7; ^{19}F NMR ($CDCl_3$) δ -67.24 (t, J = 11.0 Hz); MS m/z 260 (M^+); HRMS calcd for $C_{12}H_{11}F_3O_3$ 260.0660 (M^+), found 260.0659; IR (KBr) cm^{-1} 1718.26, 1687.41.

4-(4,4,4-Ttrifluorobutanoyl)benzonitrile (3g)

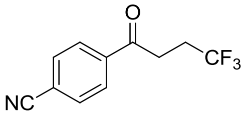

^{1}H NMR ($CDCl_3$) δ 2.62 (2H, qt, J = 10.7 Hz, 7.7 Hz), 3.26-3.30 (2H, m), 7.81 (2H, d, J = 8.5 Hz), 8.07 (2H, d, J = 8.2 Hz); ^{13}C NMR ($CDCl_3$) δ 28.2 (q, J = 30.0 Hz), 31.7 (q, J = 2.5 Hz), 116.9, 117.6, 126.8 (q, J = 275.2 Hz), 128.4, 132.6, 138.8, 194.9; ^{19}F NMR ($CDCl_3$) δ -67.25 (t, J = 10.5 Hz); MS m/z 227 (M^+); HRMS calcd for $C_{11}H_8F_3NO$ 227.0558 (M^+), found 227.0549; IR (KBr) cm^{-1} 1696.09.

4,4,4-Trifluoro-1-(*p*-tolyl)butan-1-one (3h)

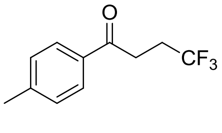

^{1}H NMR ($CDCl_3$) δ 2.43 (3H, s), 2.59 (2H, qt, J = 10.7 Hz, 7.7 Hz), 3.22-3.25 (2H, m), 7.28 (2H, d, J = 7.9 Hz), 7.87 (2H, d, J = 8.3 Hz); ^{13}C NMR ($CDCl_3$) δ 21.8, 28.5 (q, J = 29.7 Hz), 31.1 (q, J = 2.5 Hz), 127.1 (q, J = 275.2 Hz), 128.1, 129.4, 133.6, 144.4, 195.8; ^{19}F NMR ($CDCl_3$) δ -67.24 (t, J = 11.0 Hz); MS m/z 216 (M^+); HRMS calcd for $C_{11}H_{11}F_3O$ 216.0762 (M^+), found 216.0754; IR (KBr) cm^{-1} 1680.66.

4,4,4-Trifluoro-1-(4-methoxyphenyl)butan-1-one (3i)

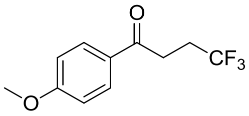

^{1}H NMR ($CDCl_3$) δ 2.58 (2H, qt, J = 10.9Hz, 7.9Hz), 3.19-3.23 (2H, m), 6.96 (2H, d, J = 8.8Hz), 7.98 (2H, d, J = 8.7Hz); ^{13}C NMR ($CDCl_3$) δ 28.5 (q, J = 29.5Hz), 30.9 (q, J = 2.5Hz), 55.5, 113.9, 127.2 (q, J = 275.2Hz), 129.1, 130.2, 163.7, 194.8; ^{19}F NMR ($CDCl_3$) δ -67.22 (t, J = 11.0Hz); MS m/z 232 (M^+); HRMS calcd for $C_{11}H_{11}F_3O$ 232.0711, found 232.0711; IR (KBr) cm^{-1} 1675.84.

4,4,4-Trifluoro-1-(3-methoxyphenyl)butan-1-one (3j)

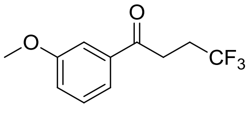

^{1}H NMR ($CDCl_3$) δ 2.59 (2H, qt, J = 10.9 Hz, 7.8 Hz), 3.23-3.27 (2H, m), 3.87 (3H, s), 7.13-7.16 (1H, m), 7.40 (1H, t, J = 8.0 Hz), 7.49-7.50 (1H, m), 7.54-7.56 (1H, m); ^{13}C NMR ($CDCl_3$) δ 28.5 (q, J = 29.7 Hz), 31.4 (q, J = 2.8 Hz), 55.6, 112.3, 120.0, 129.7, 137.4, 159.8, 196.0; ^{19}F NMR ($CDCl_3$) δ -67.24 (t, J = 11.0 Hz); MS m/z 232 (M^+); HRMS calcd for $C_{11}H_{11}F_3O_2$ 232.0711 (M^+), found 232.0705; IR (KBr) cm^{-1} 1689.34.

4,4,4-Trifluoro-1-(2-methoxyphenyl)butan-1-one (3k)

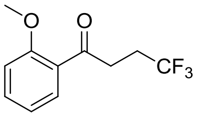

^{1}H NMR ($CDCl_3$) δ 2.55 (2H, qt, J = 1.1 Hz, 7.8 Hz), 3.24-3.28 (2H, m), 6.98-7.04 (2H, m), 7.50 (1H, td, J = 7.8 Hz, 1.5 Hz), 7.78 (1H, dd, J = 7.8 Hz, 1.9 Hz); ^{13}C NMR ($CDCl_3$) δ 28.8 (q, J = 29.5 Hz), 36.4 (q, J = 4.2 Hz), 55.7, 111.6, 120.8, 127.1, 127.2 (q, J = 274.9 Hz), 130.5, 134.0, 158.8, 198.2; ^{19}F NMR ($CDCl_3$) δ -67.22 (t, J = 11.0 Hz); MS m/z 232 (M^+); HRMS calcd for $C_{11}H_{11}F_3O_2$ 232.0711 (M^+), found 232.0703; IR (KBr) cm^{-1} 1677.77.

1-(2-Chlorophenyl)-4,4,4-trifluorobutan-1-one (3l)

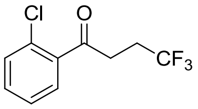

^{1}H NMR ($CDCl_3$) δ 2.59 (2H, qt, J = 10.7 Hz, 7.6 Hz), 3.22-3.26 (2H, m), 7.33-7.37 (1H, m), 7.40-7.46 (2H, m), 7.50-7.52 (1H, m); ^{13}C NMR ($CDCl_3$) δ 28.6 (q, J = 30.0 Hz), 35.5 (q, J = 2.5 Hz), 126.8 (q, J = 275.2 Hz), 127.0, 129.1, 130.7, 131.1, 133.2, 138.3, 199.1; ^{19}F NMR ($CDCl_3$) δ -67.25 (t, J = 11.0 Hz); MS m/z 236 (M^+); HRMS calcd for $C_{10}H_8ClF_3O$ 236.0216 (M^+), found 236.0194; IR (KBr) cm^{-1} 1702.84.

4,4,4-Trifluoro-1-(naphthalen-2-yl)butan-1-one (3m)

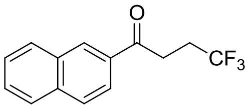

^{1}H NMR ($CDCl_3$) δ 2.65 (2H, qt, J = 10.8 Hz, 7.8 Hz), 3.38-3.42 (2H, m), 7.56-7.64 (2H, m), 7.88-7.92 (2H, m), 7.97-8.04 (2H, m), 8.48 (1H, s); ^{13}C NMR ($CDCl_3$) δ 28.5 (q, J = 29.5 Hz), 31.3 (q, J = 2.5 Hz), 123.5, 126.9, 127.1 (q, J = 274.9 Hz), 127.8, 128.6, 128.7, 129.5, 129.7, 132.4, 133.4, 135.7, 196.2; ^{19}F NMR ($CDCl_3$) δ -67.16 (t, J = 10.5 Hz); MS m/z 252 (M^+); HRMS calcd for $C_{14}H_{11}F_3O$ 252.0762 (M^+), found 252.0766; IR (KBr) cm^{-1} 1682.59.

4,4,4-Trifluoro-1-(pyridin-3-yl)butan-1-one (3n)

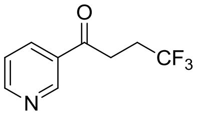

^{1}H NMR ($CDCl_3$) δ 2.63 (2H, qt, J = 10.7 Hz, 7.6 Hz), 3.26-3.30 (2H, m), 7.49 (1H, dd, J = 7.8 Hz, 4.9 Hz), 8.28 (1H, d, J = 7.8 Hz), 8.89 (1H, d, J = 2.0 Hz), 9.21 (1H, s); ^{13}C NMR ($CDCl_3$) δ 28.1 (q, J = 30.0 Hz), 31.6 (q, J = 2.7 Hz), 123.9, 126.9 (q, J = 276.4 Hz), 131.5, 135.3, 149.5, 154.0, 195.2; ^{19}F-NMR ($CDCl_3$) δ -37.25 (t, J = 10.5 Hz); MS m/z 203 (M^+); HRMS calcd for $C_9H_8F_3NO$ 203.0558 (M^+), found 203.0554; IR (KBr) cm^{-1} 1692.33.

(*E*)-4,4,4-Trifluoro-1-(4-methoxyphenyl)but-2-en-1-ol (4i)

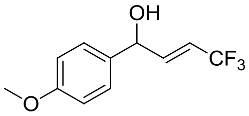

^{1}H NMR ($CDCl_3$) δ 3.84 (3H, s), 4.73-4.76 (1H, m), 5.90 (1H, dq, J = 1.9Hz, 6.5Hz), 6.38 (1H, dq, J = 2.1Hz, 15.6Hz), 6.93 (2H, d, J = 8.8Hz), 7.15 (2H, d, J = 8.8Hz); ^{13}C NMR ($CDCl_3$) δ 55.4, 75.8, 114.4, 117.5 (q, J = 33.9Hz), 123.1 (q, J = 269.1Hz), 128.8, 129.4, 140.2 (q, J = 6.4Hz), 159.9; ^{19}F NMR ($CDCl_3$) δ -64.67 (d, J = 4.0Hz); MS m/z 232 (M^+); HRMS calcd for $C_{11}H_{11}F_3O$ 232.0711, found 232.0710; IR (KBr) cm^{-1} 3420.14.

Structures of 5 and 7 obtained from computational calculation

A computational calculation was performed using Gaussian 03W at the B3LYP/6-31+G* level of theory. The calculation indicated that intermediate **5** was stabilized to the extent of 0.417 kcal/mol compared with **7**. The structures of **5** and **7** provided by the calculation are presented as a stabilized form.

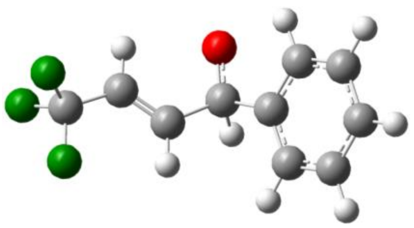

5

(Alkoxide form)

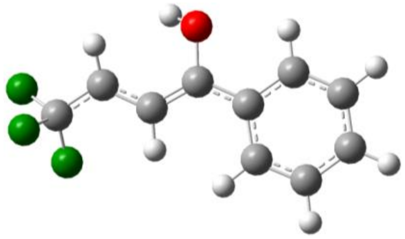

7

(Carbanion form)
